# Necdin, a p53-Target Gene, Is an Inhibitor of p53-Mediated Growth Arrest

**DOI:** 10.1371/journal.pone.0031916

**Published:** 2012-02-15

**Authors:** Julie Lafontaine, Francis Rodier, Véronique Ouellet, Anne-Marie Mes-Masson

**Affiliations:** 1 Centre de recherche du Centre hospitalier de l'Université de Montréal and Institut du cancer de Montréal, Montréal, Québec, Canada; 2 Département de radiologie, radio-oncologie et médecine nucléaire, Université de Montréal, Montréal, Québec, Canada; 3 Département de médecine, Université de Montréal, Montréal, Québec, Canada; Roswell Park Cancer Institute, United States of America

## Abstract

*In vitro*, cellular immortalization and transformation define a model for multistep carcinogenesis and current ongoing challenges include the identification of specific molecular events associated with steps along this oncogenic pathway. Here, using NIH3T3 cells, we identified transcriptionally related events associated with the expression of Polyomavirus Large-T antigen (PyLT), a potent viral oncogene. We propose that a subset of these alterations in gene expression may be related to the early events that contribute to carcinogenesis. The proposed tumor suppressor Necdin, known to be regulated by p53, was within a group of genes that was consistently upregulated in the presence of PyLT. While Necdin is induced following p53 activation with different genotoxic stresses, Necdin induction by PyLT did not involve p53 activation or the Rb-binding site of PyLT. Necdin depletion by shRNA conferred a proliferative advantage to NIH3T3 and PyLT-expressing NIH3T3 (NIHLT) cells. In contrast, our results demonstrate that although overexpression of Necdin induced a growth arrest in NIH3T3 and NIHLT cells, a growing population rapidly emerged from these arrested cells. This population no longer showed significant proliferation defects despite high Necdin expression. Moreover, we established that Necdin is a negative regulator of p53-mediated growth arrest induced by nutlin-3, suggesting that Necdin upregulation could contribute to the bypass of a p53-response in p53 wild type tumors. To support this, we characterized Necdin expression in low malignant potential ovarian cancer (LMP) where p53 mutations rarely occur. Elevated levels of Necdin expression were observed in LMP when compared to aggressive serous ovarian cancers. We propose that in some contexts, the constitutive expression of Necdin could contribute to cancer promotion by delaying appropriate p53 responses and potentially promote genomic instability.

## Introduction

Carcinogenesis is a multistep process defined by uncontrolled cell growth and neoplastic progression leading to invasive tumors and metastasis. Cancer progression models dictate that normal cells undergo a variety of genetic/epigenetic alterations which can be summarized *in vitro* by two major phenotypic changes: immortalization and transformation. Normal cells need to overcome cell cycle checkpoints and their limited division potential to achieve immortalization. Interlaced with this process, additional events contribute to cellular transformation and move cells toward the complete neoplastic phenotype [1]. Human lung and colon cancers, genetically altered mice, mouse and human cell culture models, have all been extensively used to support the multistep progression model [2], [3], [4].

Normal human epithelial or fibroblast cell transformation can be obtained with the sequential expression of a series of oncogenes, often including the viral proteins SV40LT (simian virus 40 Large-T antigen) or adenovirus early protein E1A [5], [6]. Some E1A domains conserved in SV40LT, including the CR1/CR2 Rb family (pRb's) binding domains and the p300/400-binding pocket are absolutely required for this transformation process [7]. Despite the importance of these domains, the characterization of other viral oncogenic domains involved in transformation remains incomplete and additional activities could contribute to the carcinogenesis process.

Polyomavirus (Py), an oncogenic member of the papovaviruses, causes tumors in rodents and transforms primary cells in culture [8]. In Py-induced carcinogenesis, Large-T antigen (PyLT) is responsible for inappropriate cell cycle promotion and immortalization of mouse primary cells in culture [9], [10]. This ability is mediated principally through the binding and inactivation of pRb's by the CR1/CR2 amino-terminal domains [11], [12]. PyLT genetically and functionally shares extensive homology with the closely related SV40LT, although critical differences exist. As an example, while both proteins can bind p300 and inactivate the pRb family of tumor suppressors, only SV40LT can bind and inactivate p53 [13]. Functionally, SV40LT is a dual oncogene able to immortalize and transform primary rodent cells as a single event while PyLT appears limited to immortalization *in vitro* [14]. Thus, differences between PyLT and SV40LT render these LT-Ags useful in studying different aspects of oncogenesis.

Congruent with its *in vitro* activity, PyLT drives tumor formation when expressed under various promoters in transgenic mouse models, but the lower frequency and longer latency suggest a requirement for additional secondary events [15], [16], [17]. While PyLT alone cannot transform cells in culture, it can confer resistance to growth arrest in low serum condition [10] and protect cells against Fas and TNF-α induced apoptosis [18]. This ability to evade apoptotic signals could potentially promote growth and allow cells to evade cellular-mediated immunity; important events in multistep carcinogenesis [2], [19], [20]. Moreover, while PyLT does not bind p53 directly, it has the ability to overcome some effects of this master tumor suppressor, notably p53-induced cell cycle arrest [21], [22], [23]. Finally, all E1A domains known to be essential to human cell transformation are not only conserved in SV40LT but are also found in PyLT [7]. Based on this evidence, we hypothesized that, in addition to its immortalizing activity, PyLT also modulates important functions in early mouse cell transformation.

Here, we present a strategy where PyLT induced immortalization-independent events can be revealed using NIH3T3 immortal mouse embryonic fibroblasts which already harbor immortalization-associated events that have occurred prior to PyLT introduction. Using gene expression microarray analysis, we identified Necdin among a set of genes that were consistently upregulated following PyLT expression in NIH3T3 cells. Necdin was first identified as a neuronal differentiation marker associated with growth arrest [24], [25], [26], but has since been found in several normal tissues [27], [28], [29], [30], [31]. Necdin interacts with the viral oncoproteins SV40LT and E1A [32] and is functionally similar to pRb as it can promote growth arrest by interacting with E2F1 to repress its transcriptional activity [32], [33]. In accordance with this function, Necdin overexpression shows growth inhibitory properties in NIH3T3 and SaOS cell lines [26], [32]. However, it is also expressed in myogenic precursors that have a high proliferating potential [34]. Necdin is a p53 target gene and physically interacts with the p53 protein product suggesting a functional relationship [35], [36]. Furthermore, the expression of Necdin can protect cells from apoptosis in different models [29], [33], [34], [37], [38], [39], including p53-induced apoptosis [35]. Therefore we hypothesize that during carcinogenesis, and depending on the cellular context, Necdin possesses opposing functions and may act as a tumor suppressor based on its similarity with pRb proteins, or as an oncogene through its capacity to inhibit apoptosis and p53-dependent tumor suppressive cell fates.

Results reported here support this dual functionality for Necdin. We show that despite the growth suppressive functions of Necdin, it was possible to derive growing cell populations expressing constitutively high levels of Necdin. These high levels of Necdin interfered with p53 activity and contributed to an ineffective growth arrest in response to stress. Overall, we provide evidence suggesting that upregulation of Necdin expression could provide advantages for p53 wild type cells during early carcinogenesis through its ability to decrease signaling from p53 pathways. Interestingly, we found higher Necdin expression to be associated with low malignancy potential (LMP) ovarian tumors, where p53 mutations are rare, compared to high grade invasive ovarian cancers (TOV).

## Results

### Gene statement mapping of PyLT expressing mouse fibroblasts

**Figure 1:**
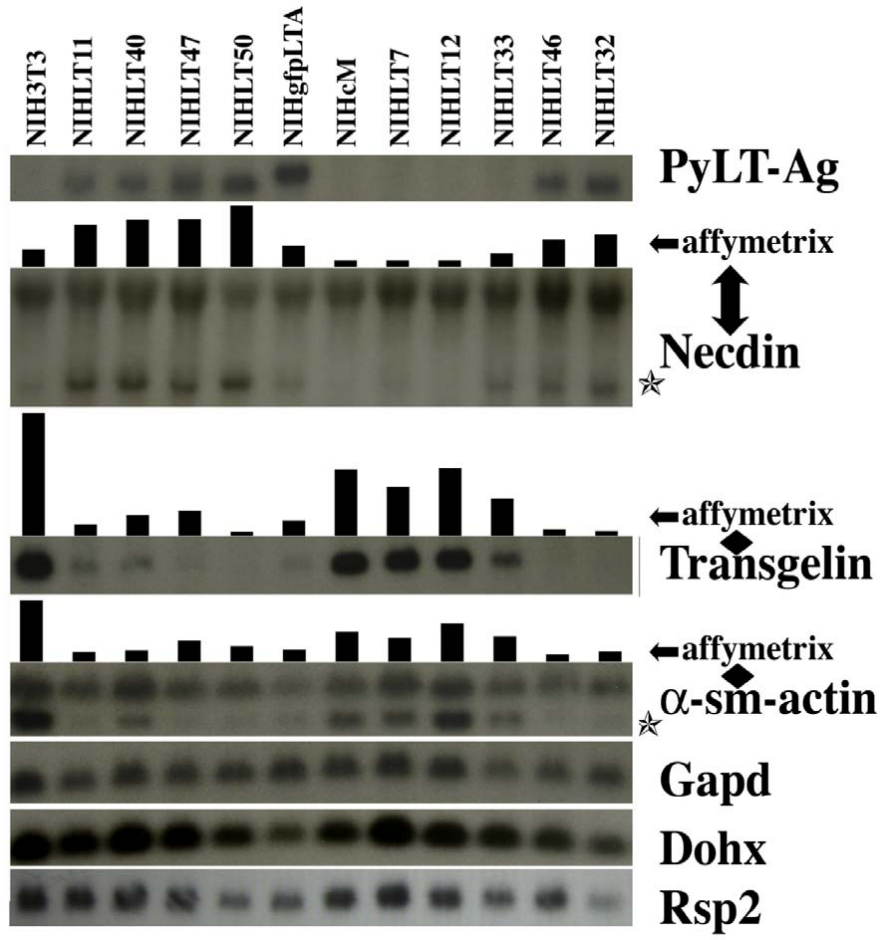
Validation of Affymetrix microarray data for selected genes in NIH3T3 and NIHLT clones. Northern blotting (using full-length radiolabeled cDNA probes) was used to validate selected Affymetrix microarray patterns. Northern blots are compared to bar graphs representing normalized microarray data. Stars represent the transcript corroborating microarray data when more then one band was detected by autoradiography for a single probe. Note the lack of detectable PyLT in clone NIHLT33, although transcripts can be seen on longer exposures (data not shown). There is no microarray data for the PyLT transcript. PyLT expression was verified on the total RNA used for microarray analysis.

NIH3T3 mouse fibroblasts were transfected with a PyLT expression plasmid and the selected clones were assessed for stable PyLT expression at the mRNA and protein level (Figure 1, Figure S1A). Selected clones were used for microarray analysis comparing PyLT-expressing clones to a second group composed of parental NIH3T3 cells as well as clones that did not express a detectable amount of PyLT. A variation cut-off set to >1.5-fold with a *P* value of ≥0.02 generated 194 candidate genes significantly modulated by PyLT, composed of 160 upregulated and 34 downregulated genes (Table S1). To refine candidate selection, we imposed a further selection criteria on the 194 genes selected in the primary analysis based on the observation that genes displaying co-transcriptional regulation often interact by functional relationships [40]. Levels of PyLT were correlated to the amplitude of fold-change expression (either up- or down-regulation) which identified 26 candidate genes whose expression varied proportionally to PyLT (Table S2). As an example, note the correlation between the variation in Necdin gene expression and PyLT (Figure S1B). These genes represented the strongest candidates with 15 upregulated and 11 downregulated genes.

The generated data was validated by Northern blot analysis using a selected number of genes. Expression levels on Northern blots were compared to corresponding microarray expression values (Figure 1, and data not shown). Gene expression variations observed on Northern blots with full length cDNA probes were highly similar to the data generated by the Affymetrix oligonucleotide microarray for all probes and clones tested (Figure 1). Some full-length cDNA probes generated more than one band when using radioactive Northern blots although at least one band of the expected molecular size closely followed the microarray pattern (see α-sm-actin and Necdin, Figure 1). In addition to loaded RNA quantification, Gapd, Dohx as well as Rsp2 showed little variation across all clones and were used as loading controls.

### Validation of a selected candidate gene, Necdin, on an extended NIH3T3 clone set

**Figure 2:**
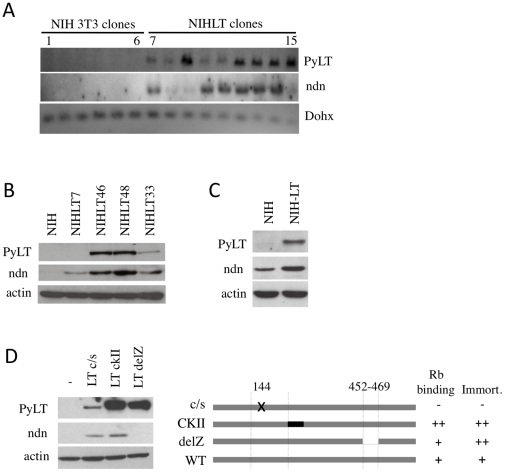
Necdin as a relevant candidate. (A) Validation of the microarray identified Necdin gene by Northern blot analysis on an independent extended clone set. Lanes 1 to 6 represent individual NIH3T3 sub-clones. Lanes 7 to 15 represent individual NIH3T3 clones transfected and selected to express PyLT. Clones were independent from those used in the microarray analysis. DIG-labeled probes were used and exposure times were adjusted. DOHX was used as a control. (B–C) Necdin protein level in (B) clones expressing variable level of PyLT or (C) additional heterogeneous populations of NIH3T3 cells stably transfected with PyLT (NIHLT) or empty vector (NIH). (D) Different mutant forms of PyLT protein expressed in NIH3T3 were used to determine the domain involved in Necdin modulation. Western blot shows protein expression levels. Representation of mutants utilized with Rb-binding and immortalization capacity as reported previously [12], [41].

**Figure 3:**
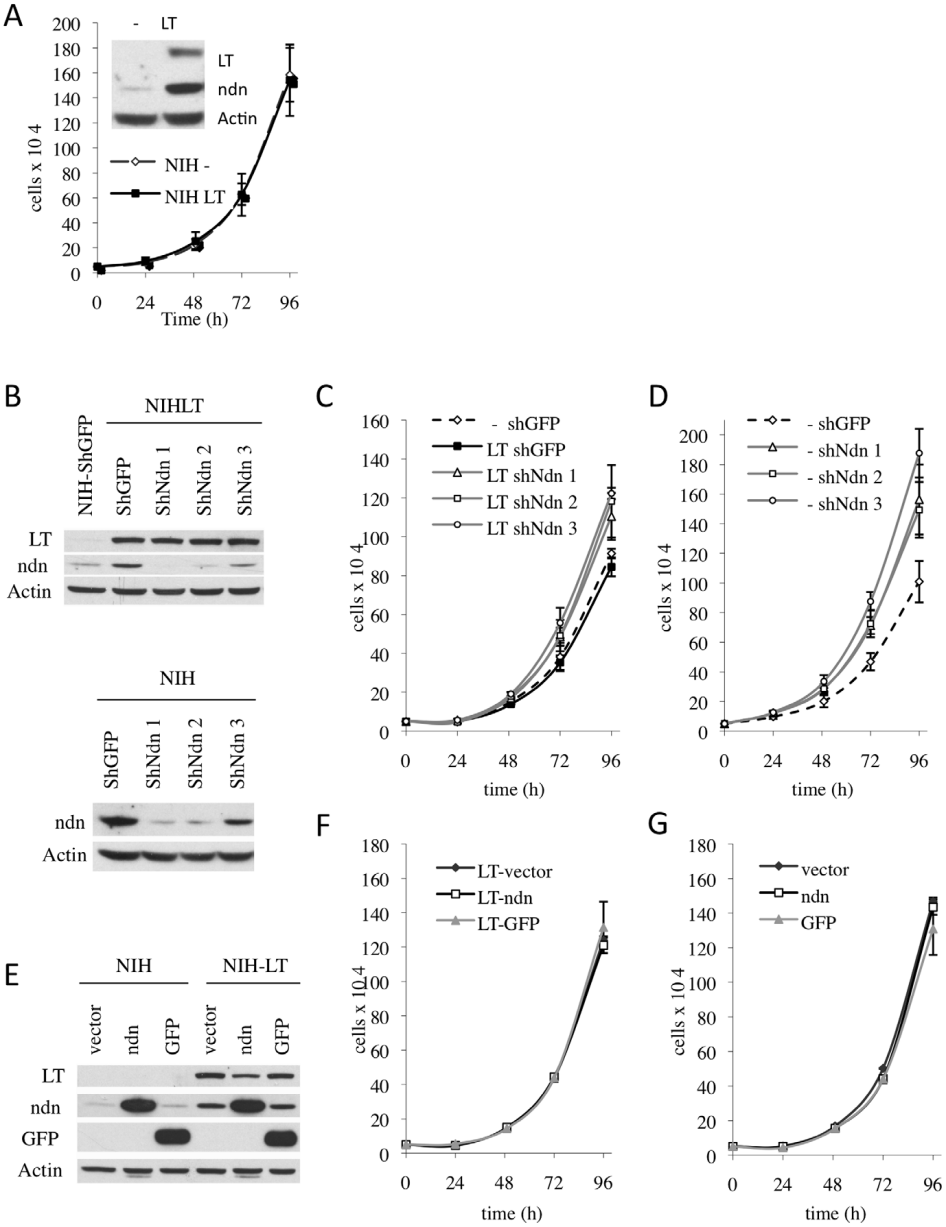
Necdin influences the proliferation of NIH and NIHLT populations. (A) NIH and NIHLT mixed populations proliferate at the same rate. (B) Decrease of Necdin protein levels by three different shRNAs transduced by lentivirus infection in NIHLT and NIH. Growth curves for NIHLT (C) and NIH (D) revealed that cells depleted in Necdin proliferate faster. (E) Protein expression level in NIHLT and NIH cells with overexpression of mouse Necdin, the GFP control or vector alone. Growth curves for NIHLT (F) and NIH (G) expressing exogenous Necdin after a certain period of time did not shown differences in their growth rate.

Among all candidates identified, the gene encoding Necdin was selected for further study. Microarray analysis showed an upregulation of mRNA up to five-fold (T-test, P<0.001) (Table S2 and Figure S1B). In addition, a second probe set was associated with the Necdin gene (94811_s_at) and also revealed a 3.6-fold upregulation, although with a P value of 0.04 (data not shown). To further validate the microarray data, Necdin expression was analyzed on an extended set of six NIH3T3 sub-clones and nine independent PyLT-expressing NIH3T3 stable clones not included in our initial analysis (Figure 2A). The higher expression levels of Nectin observed when PyLT is expressed, as determined by Northern blot analysis, correlated well with the data derived from microarray analyses. Moreover, a nonradioactive Dig-labeled probe gave only one specific band around the expected size of 1.6 kb, confirming the identity of the lower band in Figure 1 as Necdin. Some clones with variable levels of PyLT expression were also used to confirm that the variation measured at the RNA level was reproduced at the protein level for Necdin (Figure 2B). Furthermore, when we derived a new heterogeneous population of NIH3T3 cells expressing PyLT (NIHLT), we again observed an upregulation of Necdin expression compared to a vector-transfected population control (NIH) (Figure 2C and Figure 3A inset). Necdin variation could be seen as early as 72 hrs post-transfection of PyLT. These results show that elevated Necdin expression levels were a reproducible and constant phenotype in PyLT-expressing NIH3T3 cells and not caused by a clonogenic effect, thus suggesting that Necdin may be involved in some of PyLT oncogenic functions.

### PyLT induced Necdin expression independently of Rb inactivation

In order to understand the underlying mechanisms behind the increase in Necdin expression upon PyLT expression, we used different well-characterized mutants of important PyLT domains (Figure 2D). Rb binding deficient LT-c/s is unable to immortalize primary cells while LT-CKII has an increased Rb binding capacity compared to wild-type and demonstrates twice the immortalization potential [12]. Deletion of the zinc finger in mutant LT-delZ does not affect Rb binding but enhances the immortalization potential of the protein [41]. The amount of protein expressed from transfection of these three mutants in NIH3T3 cells was variable, with the mutant LT-c/s exhibiting the lowest level of expression (Figure 2D). However, it was clear that the LT-delZ mutant, even with a high level of expression, did not induce Necdin when compared to other mutants. The LT-c/s mutant was able to reproduce the increase in Necdin expression even with an overall lower protein level. The LT-CKII mutant also showed similar increases in Necdin expression (Figure 2D).

These results suggest that Necdin expression is not directly associated with the immortalization function of PyLT since the mutation of PyLT affecting the modulation of Necdin expression (LT-delZ), did not compromise its immortalization potential.

### Necdin overexpression does not affect NIH3T3 proliferation in long term experiments

Necdin has been reported as a growth suppressor [26], and it is counterintuitive that its expression would be stimulated by a viral protein such as PyLT whose main function is to stimulate cell cycling to promote viral DNA replication [9], [42]. NIH3T3 and PyLT-expressing NIH3T3 cells were thus compared. No differences in cell proliferation were observed (Figure 3A).

To further assess the effect of Necdin modulation in our model, we used either gain or loss of function experiments. Three different shRNAs (shNdn1–3) were transduced in NIH and NIHLT cell populations resulting in decreased Necdin expression (Figure 3B). Consistent with a role for Necdin as a growth suppressor, removing Necdin expression by shRNA increased cellular proliferation of NIH and NIHLT cell populations (Figure 3C and 3D). Additionally, no cell death was observed in NIHLT cells after Necdin removal indicating that its expression was not necessary to maintain a PyLT-expressing cell population. While NIH and NIHLT cells proliferate at the same rate, it remained possible that Necdin levels were not elevated enough to cause growth arrest in our cell lines. Therefore, we overexpressed Necdin in NIH and NIHLT cells by using a lentiviral transduction system (Figure 3E). A transient decrease in growth rate was observed shortly after the expression of Necdin (Figure S2), as previously shown by others [26]. However, maintaining these populations for longer periods of time in culture rapidly allowed us to derive populations that still expressed high levels of Necdin without any growth defects (Figure 3F and Figure 3G). These populations stably expressing Necdin were further characterized.

### PyLT allows bypass of p53-dependent growth arrest induced by nutlin-3

**Figure 4:**
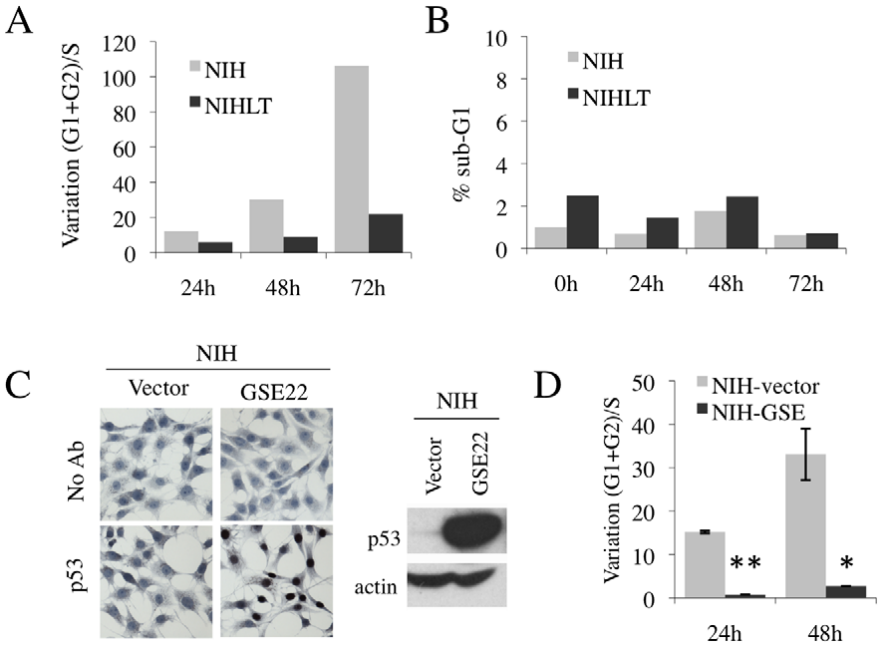
Nutlin-3 induces a p53-dependent growth arrest in NIH3T3 cells that is bypassed by PyLT expression. (A–B) Flow cytometry analysis of NIH or NIHLT populations treated with nutlin-3 (5 µM) demonstrate that nutlin-3 induces a growth arrest in NIH cells, but not in NIHLT cells. Results presented are from one representative experiment (A) Cell cycle arrest was represented by the variation of ratio of arrested cells (G1+G2 phases) over proliferating cells (S phase) in treated cells versus untreated controls. (B) No variation of the percentage of cells in Sub-G1 phase, representing cell death, was observed after nutlin-3 treatment. (C–D) The use of a p53 inhibitor peptide (GSE22) validates the p53-dependence of the growth arrest induced by Nuclin-3. (C) High efficiency of infection and functionality of the GSE22 peptide were demonstrated by the accumulation of non-functional p53 in the nucleus by immunocytochemistry detecting p53 in NIH transduced with GSE22 or control vector. The stabilization of non-functional p53 was also seen in Western blots of the corresponding infected cells. (D) FACS analysis on NIH transduced with GSE22 or vector with nutlin-3 treatment (*P<0.05, **P<0.01 t-test).

Necdin interacts with p53 and possibly modulates its activity [35], [39], which raises the possibility that PyLT exerts its inhibitory effect on p53 through Necdin induction. Nutlin-3 is a small molecule antagonist of MDM2, which prevents the interaction between MDM2 and p53, thus promoting the accumulation of p53 in cells [43]. It has been recently shown that nutlin-3 can efficiently induce cell cycle arrest or apoptosis in different cancer cell lines with functional p53 [44]. To assess the response induced in our model, the NIH3T3 cell line was treated with nutlin-3 and proliferation was followed by flow cytometry. Stimulation of NIH cells with nutlin-3 resulted in the stabilization of p53 causing p21 induction (Figure S3A) and a gradual growth arrest (Figure 4A). We did not detect apparent cell death as evaluated by the sub-G1 content (Figure 4B). When PyLT-expressing NIH3T3 cells were treated with the same dose of nutlin-3, we observed an important delay in growth arrest without a significant elevation in the amount of cell death (Figure 4A and 4B). To confirm that growth arrest obtained in our model was actually dependent on p53, we used a dominant-negative p53 peptide, GSE22 [45], [46], delivered by lentivirus. As revealed by immunostaining, high infection efficiencies were reached with lentiviruses since almost all cells showed expression of GSE22, which resulted in an accumulation of non-functional p53 in the nucleus (Figure 4C). Inactivation of p53 by GSE22 expression (NIH-GSE22) conferred almost complete resistance to nutlin-3 (Figure 4D) thereby showing the p53-dependence of nutlin-3 induced cell cycle arrest in NIH3T3 cells. These results show that PyLT expression clearly protects against a p53-dependent growth arrest, which supports previous reports on the inhibitory activity of the viral protein on p53 [21], [22].

### p53-dependent growth arrest is delayed by Necdin

**Figure 5:**
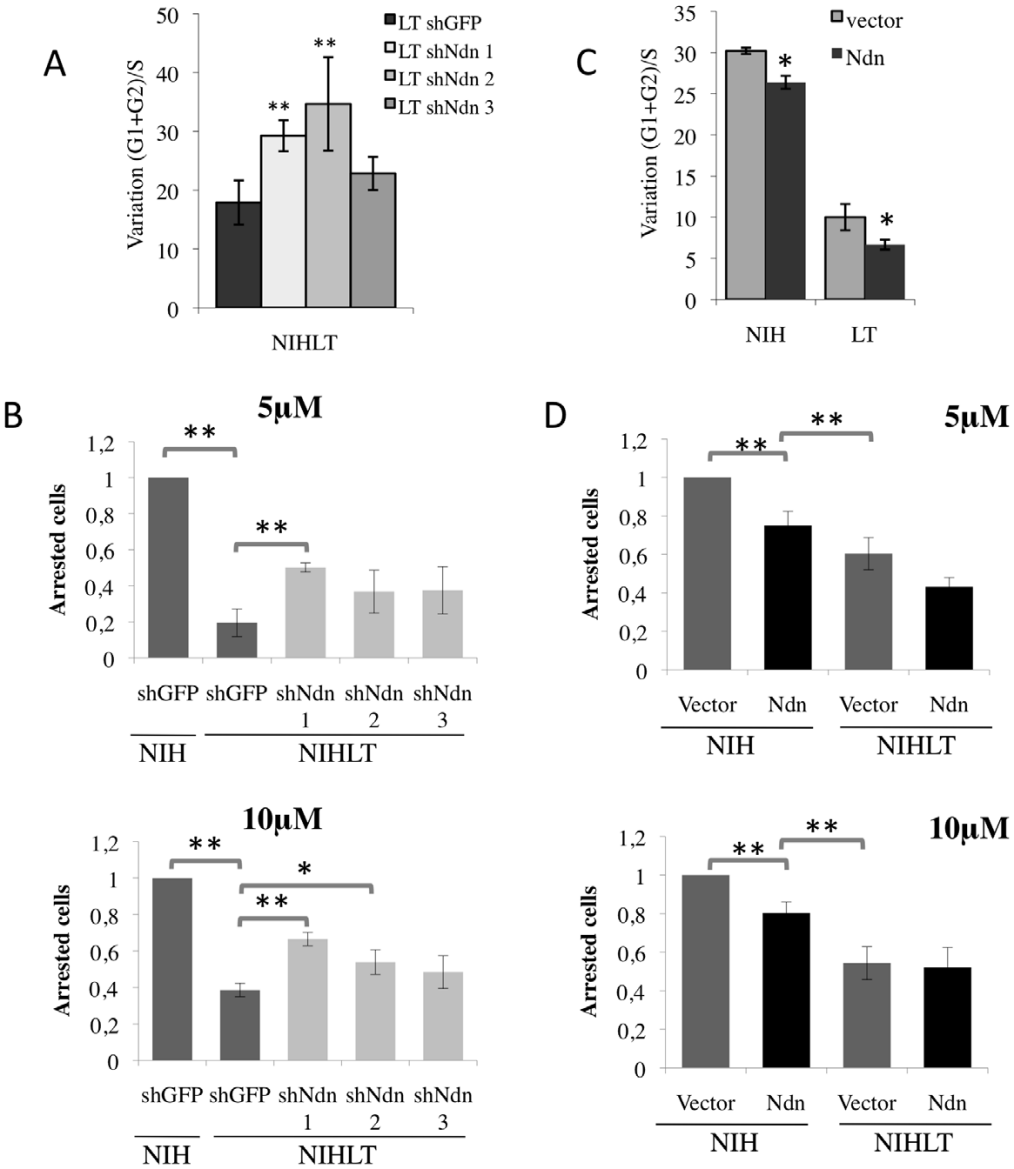
Necdin confers resistance to p53-dependent growth arrest. (A–B) NIHLT cells depleted in Necdin by shRNAs and exposed to nutlin-3 showed an increase in growth arrest (A) measured by DNA content analysis by flow cytometry (as described in figure 4A) or (B) assessed by Wst-1 colorimetric assay. Results for Wst-1 represent normalized data according to the portion of arrested cells (O.D. untreated – O.D. treated) relative to arrested control NIH after 48 h of exposure to nutlin-3. (C–D) NIH and NIHLT cells overexpressing Necdin showed growth arrest resistance upon nutlin-3 treatment. (C) FACS analysis or (D) Wst-1 colorimetric assay (*P<0.05, **P<0.01 t-test).

To address whether the presence of elevated Necdin levels in PyLT expressing cells may play a role in the delayed p53-response, we examined cell cycle distribution upon nutlin-3 treatment in cells where Necdin expression was decreased by the use of three different shRNA (Figure 3B). In response to nutlin-3 treatment for 48 hours, an increase in cell cycle arrest was observed when suppressing Necdin expression in NIHLT cells compared to NIHLT infected with the control recombinant virus, shGFP (Figure 5A). It was observed that shNdn 3, which repressed Necdin less efficiently (Figure 3B), only showed a limited effect (Figure 5A). Thus, the reduced presence of Necdin in NIHLT cells sensitized them to p53 cell cycle arrest. We did not notice significant changes using flow cytometry assays in NIH cells expressing shNdn constructs presumably due to the fact that the parental cells already expressed very low levels of Necdin, and were already highly sensitive to cell cycle arrest (Figure S3B).

To validate these results, we also used Wst-1 assays to assess the effect of Necdin loss on cell growth. Again, reduction of Necdin levels by shRNA sensitized NIHLT to cell proliferation arrest induced by nutlin-3 (Figure 5B). Significant changes where observed for shNdn 1 and 2 at a dose of 10 µM while shNdn 3 did not vary significantly. In all experiments, targeting Necdin in NIHLT did not convey the same sensitivity as NIH cells (Figure 5B). Unlike results obtained using flow cytometry, reduction of Necdin levels in NIH cells did sensitize them further to the p53-induced growth arrest when measured using the Wst-1 assay (Figure S4C).

Conversely, Necdin overexpression delayed p53-mediated growth arrest both in NIH and NIHLT as evaluated by DNA content (Figure 5C). Consistent with flow cytometry, Wst-1 assays revealed that the ectopic expression of Necdin appeared to attenuate the effect of nutlin-3 in NIH and NIHLT (but only at the 5 µM concentration for NIHLT), although this reached statistical significance only in NIH cells (Figure 5D). It should be noted that the mere overexpression of Necdin did not confer to NIH cells the equivalent response to nutlin-3 seen in the NIHLT cells (Figure 5C and 5D). These results suggest that the acquired resistance to growth arrest in PyLT-expressing NIH3T3 cells was in part mediated by Necdin expression but also that other factors were presumably involved. Nevertheless, Necdin could confer growth arrest resistance even in the absence of PyLT.

### Necdin is a p53-target gene induced by different genotoxic stress

**Figure 6:**
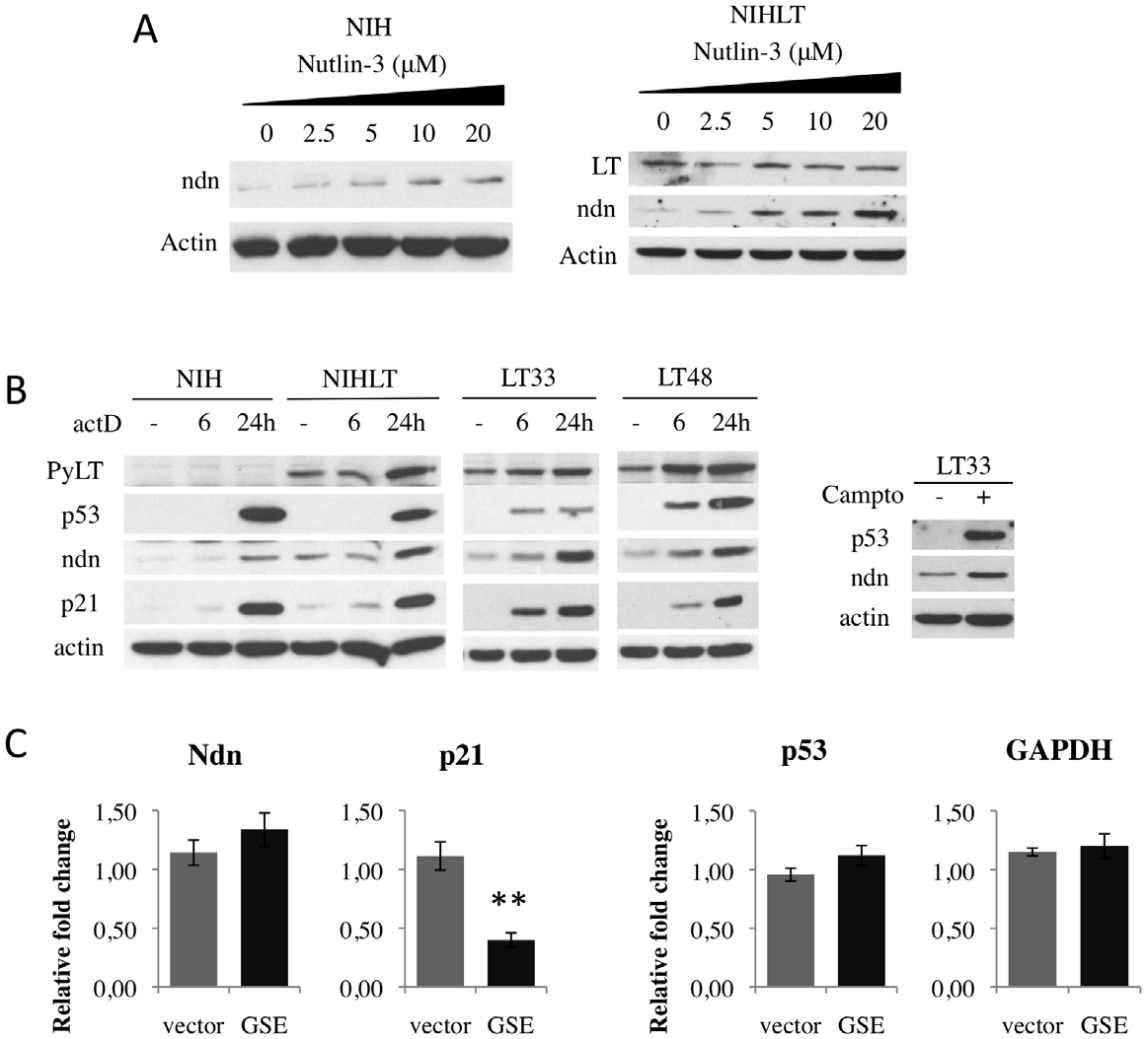
PyLT-induced Necdin expression is p53-independent. Necdin is induced following activation of p53. (A) Dose response treatment with nutlin-3 increased Necdin protein level in NIH (left) and NIHLT (right). (B) Genotoxic stress induced by Actinomycin D and Camptothecin also stimulated Necdin protein expression. (C) In NIHLT cells, Necdin expression is not dependent on p53 activity as assessed by p53 inhibition. Mean of relative expression of Necdin, p21, p53 and GAPDH in NIHLT cells with or without the p53 inhibitor GSE22. Expression was measured by Q-PCR in three independent samples from each group. Expression is relative to actin (**P<0.001, t-test).

As shown in Figure 6A, a dose-dependent elevation of Necdin protein levels in NIH and NIHLT cells were observed after exposure to nutlin-3. This increase was also observed at the RNA level (Figure S4A and S4B) suggesting transcriptional regulation, !?tlsb=-.005w?>rather than a protein stabilization. This transcriptional regulation was p53-dependent since inactivation of p53 with GSE22 peptide abolished the Necdin increase seen in response to nutlin-3 stimulation (Figure S4C). This is consistent with a recent report suggesting that Necdin is a direct target gene of p53 [36]. We then examined if other genotoxics stresses known to induce p53 could also cause Necdin upregulation. Both Camptothecin, a Topoisomerase I inhibitor, and Actinomycin D, an inhibitor of transcription, are known to induce p53 activation [47]. Increased Necdin expression levels were observed in all cells treated with these two drugs (Figure 6B). By extension, this suggests that Necdin is a part of p53 pathways that can be induced by different signals.

### Necdin upregulation in PyLT-expressing cells is independent of p53

Since p53 activation induces Necdin expression, we hypothesized that the PyLT-mediated induction of Necdin may involve an increase in p53 basal activity in NIHLT cells. To assess p53 involvement, the p53 inhibitor GSE22 was introduced in NIHLT cells and changes in p53-induced gene expression were analyzed. Expression of GSE22 affected basal mRNA of p53 target genes as judged by the level of p21 (2.5-fold decrease) but did not affect the control GAPDH (Figure 6C). In the same extract, the Necdin level was not affected by inhibition of the p53 function (Figure 6C). These data demonstrate that although Necdin is induced by p53 in response to cellular stresses, PyLT does not require p53 to mediate increased Necdin expression levels.

### Necdin is expressed in low malignant potential ovarian cancer

**Figure 7:**
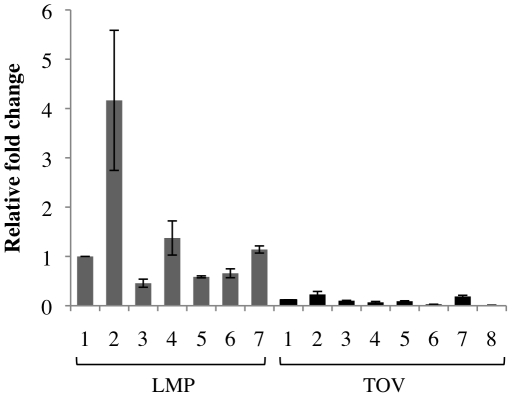
Necdin expression is detected in LMP and is lower in TOV. Q-PCR analysis of tissues from seven LMP serous ovarian cancers and eight high grade serous ovarian cancers. Expression of Necdin gene (*P*<0.0001, Mann-Whitney's U test) relative to ERK-1.

Since Necdin expression can be regulated independently of p53 and can repress p53 activity, it is possible that deregulation of Necdin may be important in cancers with wild type p53, where Necdin may play a role in inhibiting the p53 tumor suppressor activity. To test this, we examined Necdin expression in low malignancy potential (LMPs) versus aggressive ovarian cancer (TOVs), two distinct types of serous ovarian cancer thought to have different molecular origins, and where p53 mutations rarely (in LMPs) or frequently (in TOVs) occur [48], [49]. A set of seven LMP and eight TOV tissues were used and Necdin mRNA levels were determined by quantitative real-time PCR analysis. The relative expression levels revealed a significant difference in Necdin expression (*P*<0.0001) between tissues from LMP and TOV, with higher levels found in LMP tissues (mean value was >10-fold higher) (Figure 7).

## Discussion

Genes regulated by PyLT were identified in a mouse fibroblast cell culture model. Considering that PyLT has anti-apoptotic activities [18], that it maintains strong homologies in essential domains to the transforming oncogenes SV40LT and E1A [7], and that its expression in transgenic mice leads to tumors development [15], [16], it was hypothesized that these PyLT structure-function properties could provide clues to early steps during the transformation process. Since NIH3T3 cells were already immortalized mostly through the biallelic deletion of the INK4 locus [50], [51], PyLT-mediated immortalization was not a selection criteria in our model and we considered candidate genes as possibly immortalization-independent.

Our microarray analysis identified a list of potential transformation-associated candidate genes that corroborates the existing literature and point out the importance of viral proteins as tools to identify events related to cancer progression. For example, Transgelin, an actin-binding protein downregulated in our study, is also downregulated in virally transformed human cells and in human breast, colon and lung cancers [52], [53]. Alternatively, DNA methyltransferase 1 (Dnmt1), which contributes to the maintenance of tumor suppressors silencing in colon cancer progression and in tumorigenic cell lines [54], [55], is also upregulated by PyLT expression. Importantly, Dmnt1 is recognized as a significant event during the carcinogenesis process in models related to polyomavirus T antigen expression including the prostate cancer mouse model expressing SV40LT (TRAMP) [56], and transformation of cell lines by SV40LT or the human polyomavirus BKV [57], [58]. Interestingly our main candidate gene, Necdin, was also upregulated in a mouse prostate cancer progression model based on SV40LT expression [59]. Initial observations for Necdin expression in human cancer suggested a tumor suppressor function due to its lack of expression in brain tumor cell lines [25], its decrease in melanomas [60], and in bladder cancer cell lines and tumors [61]. Conversely, more recent studies revealed loss of imprinting and upregulation of Necdin in pancreatic cancer [62], [63]. As a whole, Necdin function in cancer remains poorly defined and warrants further investigation.

### Identification of functional activities associated with PyLT

One way to identify closely interacting proteins (physical or functional associations) is to monitor their mRNA expression levels since they are often co-regulated [40]. Since the group of genes shown in Table S2 most closely correlates with PyLT expression at the transcriptional level, these genes represent good candidates for functional associations. One particularly promising member of this group is Necdin, whose gene product has Rb-like growth regulatory activities and has been shown to interact with p53 and viral oncogenes such as SV40LT and E1A [32], [35]. It has been hypothesized that the Rb-like activity of Necdin leads to cell growth arrest when overexpressed in neurons and fibroblasts [26], [32]. In particular, the growth inhibitory effects of Necdin were described in a model system using NIH3T3 cells [26]. Here, we demonstrate that PyLT expression in NIH3T3 cells results in increases in both Necdin transcript and protein levels but without altering the long-term growth of PyLT-expressing cells. This suggests that PyLT can inhibit the known growth suppressive functions of Necdin [26]. Surprisingly, continued proliferation in the presence of high levels of Necdin was not due to the simultaneous expression of PyLT since the overexpression of Necdin alone produced long-term Necdin-expressing NIH3T3 cells. Indeed, cell growth slowed immediately following Necdin expression, as previously described [26], but as shown in this study, the cells rapidly recovered and maintained normal proliferation rates while maintaining high Necdin levels. This divergence in Necdin-influenced cell proliferation may be explained by differences between the experimental approaches. Although the previous study also used the mouse *Ndn* sequence, the protein was conditionally expressed in NIH3T3 cells with an inducible expression system. Three independent clones were selected and analyzed immediately after induction. In contrast, we used lentivirus-mediated Necdin expression and evaluated the growth potential of heterogeneous populations after a period of selection and some passages in culture. While long-term Necdin overexpression was not incompatible with cell growth, we cannot yet conclude whether the emerging population came from a selective pressure for cells able to tolerate high Necdin expression or if they came from a transient anti-proliferative effect of Necdin from which most cells can adapt. Interestingly, we also experienced difficulty with the production of recombinant lentiviruses from Necdin constructs (also reported by [32]), potentially reflecting Necdin related growth inhibition in the packaging cell line 293FT.

### Necdin regulation by PyLT-Ag

The increase in Necdin expression in response to PyLT expression was not caused by Rb inactivation but was dependent on the presence of the PyLT C-terminal zinc finger domain. This PyLT domain is required for viral DNA replication possibly due to its involvement in protein-protein interaction, which allows the formation of PyLT hexamers [64]. Given that the zinc finger motif is conserved in several polyomavirus large T antigens, this supports the idea that other large T antigens may also induce Necdin expression. Although mutations in this domain do not abolish the immortalization property of PyLT [41], deletions in this domain of SV40LT or papillomavirus E7 decreased their transforming potential [65], [66]. Accordingly, Necdin could have a potential role in the transformation process involving viral proteins but not in immortalization. Necdin induction by PyLT could arise through direct PyLT interaction with DNA or with cellular transcription factors via its zinc finger domain to enhance their activity at the Necdin promoter. One promising candidate is Lim domain only 4 (LMO4) known to control the expression of the *Ndn* gene [67] and that was also upregulated following PyLT expression (Table S2). LMO4 demonstrates variable expression in different cancers but its role remains unclear since in breast cancer, high LMO4 expression is associated with a poor prognosis [68], [69], while in pancreatic cancer it is associated with a better survival [70], [71].

### The p53-Necdin negative feedback loop

Necdin was identified in our screen using conditions that highlight stable events occuring in continuously proliferating cells. These conditions presumably reflect the plasticity of heterogeneous cancer tissue where each cell will not have the exact same fate upon exposure to stress and where selection pressures allow the emergence of growth/survival promoting properties. According to the tumor suppressor function previously proposed for Necdin, it would be beneficial for a cell to lose Necdin expression to acquire a proliferative advantage, unless maintaining Necdin was somehow advantageous to the cancer cell. Therefore, we examined whether in some contexts, increased Necdin could paradoxically promote growth or survival.

A possible role for Necdin in DNA damage response was suggested by the upregulation of Necdin following different genotoxic stresses. By using nutlin-3, we showed that p53 activation clearly induced Necdin in a dose dependent manner, supporting a previous report that identified Necdin as a p53 target gene [36]. Moreover, we show that modulation of the Necdin level affects p53-dependent growth arrest. Indeed, we demonstrate that an increase in Necdin expression results in a delayed cell cycle arrest while inversely targeting Necdin by shRNA accelerates this arrest. The interaction of Necdin with p53 [35] suggests that this delay in growth arrest is probably associated with a direct inhibitory effect of Necdin over p53. We noted that Necdin affected p21 induction following p53 activation in our model (Figure S3D and S3E) supporting previous results [35]. Therefore, interference with p53 transcriptional activity may represent the mechanism underlying the cell cycle arrest variations caused by Necdin. However, we believe that other mechanisms may be involved since p21 mediated-arrest mostly relies on functional Rb [72], [73] and in PyLT-expressing cells, the Rb proteins are kept inactive by their interaction with PyLT [74], [75].

As p53 induction upon genotoxic stress is associated with multiple additional signaling events, we directly addressed p53 stimulation by exposure to nutlin-3. This specific stimulation results in a functional induction of p53, although the post-translational phosphorylation of p53 observed with genotoxic stress are absent or barely detectable with nutlin-3 [76], [77]. This suggests that phosphorylation may not be critical for interaction of Necdin with p53 and that Necdin does not interfere with the phosphorylation status to modulate p53 activity. In addition to phosphorylation, other modifications contribute to p53 activity [78], including acetylation, which is increased upon nutlin-3 stimulation [79]. The deacetylase Sirt1 is a negative regulator of p53 activation [80], [81] and Necdin interactions with this protein potentiate its activity upon genotoxic stress [39]. However, we did not address the status of these post-translationals modifications in our model. Additionally, it is important to note that p53 responses can differ with particular drugs depending on the dose employed, the duration of the treatment, and the metabolic state of the cell [82], [83].

Others mechanisms can explain Necdin inhibitory effects over p53. Necdin binds the N-terminal transactivation domain of p53 [35]. Some proteins share this binding site, among them SOCS1, which contribute to p53 activation [84]. It is possible that Necdin competes with activating proteins such as SOCS1 for p53 binding, leading to a decrease in p53 response. Others examples exist where the response to p53 activation varies according to the presence or absence of specific cellular partners. The capacity of p53 to translocate to the mitochondria where it plays a transcription-independent function in apoptosis is now well documented. Tid1 is a p53-interacting protein that helps this localization from the nucleus to mitochondria [85]. Both cytoplasmic and nuclear cellular partners have been revealed for Necdin and expression of these partners has been shown to cause Necdin relocalisation in the cell [28], [86], [87], [88], [89]. Perhaps interference with p53 activation may arise from the ability of Necdin to relocate p53 in other cellular compartment. All these mechanisms are consistent with the notion that Necdin can inhibit p53 function and require further investigation.

Combining our data on p53 inhibition by Necdin with the knowledge that Necdin is a direct p53 response gene suggests that Necdin is part of a negative feedback loop controlling p53 activity. Under normal conditions, this loop is probably well controlled and allows normal regulation of cellular responses as in the case of the p53 negative regulator and target gene mdm2 [90], [91]. Importantly, our results also show that Necdin can be induced by PyLT in a p53-independent manner, which, in a cancer context, could cause a reduction in p53 activity and potentially contribute to checkpoint bypass and genomic instability [92].

### Necdin is expressed in the borderline ovarian cancer subtype

According to the literature, Necdin expression may not be suitable for tumor progression. Necdin has an anti-angiogenic function by interacting with HIF-1-alpha and by negatively regulating its activity on VEGF induction [93], [94]. VEGF play a major role in the proliferation and migration of endothelial cells, thereby nourishing and favoring tumor growth by a pro-angiogenic function. Moreover, Crawford and al. [95] identified some genes predictive of metastasis in breast cancer from a quantitative trait locus analysis and found Necdin among their candidates whose expression diminished with increased risk of metastasis. These results evoke a possible limited capacity of tumor progression to an advance stage in the presence of Necdin expression. In this study, we chose to further characterize Necdin in ovarian cancer since this pathology includes a particular subset of low malignancy cancer. LMPs are non-invasive, or only display micro-invasion, rarely progress to an aggressive metastatic cancer and patients with LMP disease have a 5 years survival rate of 95%. Here, we observed higher expression of Necdin in LMPs compared to TOVs. In line with this, LMPs have low angiogenesis as compared to TOVs [96], which fits nicely with Necdin's anti-angiogenic activity.

Moreover, LMP rarely have mutation in *TP53* while 50 to 80% of high-grade carcinomas present abnormalities in *TP53* [48], [49]. This could indicate that in LMPs, where p53 is wild type, alternative mechanisms are responsible to render p53 inactive. Our results revealed an inhibiting function of Necdin over p53-dependent growth arrest. Therefore, Necdin expression in LMPs may attenuate the response when p53 activity is required. Taken together, the data support the notion that in ovarian cancer, Necdin expression correlates with a favorable prognosis. The hypothesis that LMP tumors are precursors of invasive tumors is still controversial, but the evidence suggests that they are two distinct diseases. Expression of Necdin in borderline ovarian tumors could be characteristic of this particular ovarian cancer and may have a biologic impact on p53 pathways and malignancy. However, these possible functions require more investigation.

### Conclusion

While the temporal order of multi-step carcinogenesis events may not be crucial, especially since immortalization and transformation are *in vitro* concepts, the pathways or genes themselves may point to important parameters during carcinogenesis. It is likely that some of the candidate genes identified here may play a role in human cancer. Our results suggest that Necdin harbors both tumor suppressive or oncogenic properties depending on the cellular context. These oncogenic properties were demonstrated here by the inhibitory effect of Necdin over p53-mediated growth arrest and by others where Necdin contributed to p53-induced apoptosis resistance [35], [39]. In combination with Necdin expression patterns during ovarian cancer progression, these results warrant further investigation about the context-dependent oncogenic properties of Necdin. Further challenges include investigating the functional significance of the identified candidates during multistep carcinogenesis.

## Materials and Methods

### Ethics Statement

The Centre hospitalier de l'Université de Montréal (CHUM) institutional ethics committee approved the ovarian tumors study and written consent was obtained from patients prior to sample collection.

### Cell culture

NIH3T3 cell lines [97] were purchased from ATCC. All cell lines were cultured in Dulbecco's modified Eagle's media (DMEM) supplemented with 10% fetal bovine serum, gentamycin and amphotericin. Cells were grown at 37°C with 5% CO_2_ and kept at low-density conditions to prevent culture induced transformation [97], [98]. For proliferation experiments, 5×10^4^ cells were seeded in 6 well plates and the number of living cells determined using the CASY® cell counter model TT or by a hematocytometer. Experiments were repeated at least three times in duplicate. For p53 stimulation, the following reagents was used: Nutlin-3 (5–20 µM), Actinomycin D (60 µM) and Camptothecin (5 µM).

### Vectors and transfections

The PGKLTneo plasmid was constructed by introducing Neomycin resistance from the pSV2neo vector into PGKLT [18] and was used to transfect NIH3T3 cells. The PGKLTGFPneo vector was generated by cloning eGFP from Clontech eGFP-N1 vector in frame with PyLT into PGKLTneo. A small C-terminal deletion of PyLT was introduced encompassing amino acids 685–785. All stable clones were selected with 0.5 mg/ml G418 applied 48 hrs post-transfection and named “LT” follow by a different number representing each independent clone. Heterogeneous populations expressing PGKLTneo plasmid or the control vector PGKN were generated with Lipofectamine 2000 transfection reagent (Invitrogen). We referred to these heterogeneous populations as NIHLT and NIH respectively. Mutant forms of PyLT are described elsewhere [12], [41]. Briefly, LT c/s contain a cysteine to serine substitution in amino acid 144. In LT CKII, some amino acids in the phosphorylation motif adjacent to the Rb binding motif were substituted to mimic an E7 CKII motif. LT delZ contains a deletion in amino acids 452 to 469 in the zinc finger domain.

### Lentiviral constructs and infections

Mouse *Ndn* was PCR amplified from a Riken clone (clone 1500000G13) followed by insertion in pENTR/D-TOPO® (Invitrogen). The generated pENTR-ndn vector was recombined in the 670-1 vector (pLenti CMV/TO Puro DEST, Addgene 17293) [99] using recombination cloning technology from Invitrogen. Empty control vector (referred to as Vector in figures) was the product of 686-1 (pENTR4 no ccDB, Addgene number 17424) [99] recombined with the 670-1 vector. eGFP and GSE22 (encoding an interfering p53 fragment) are described elsewhere [45], [100]. For gene repression, pLKO.1 lentiviral shRNA vectors targeting the mouse *Ndn* gene were purchased from Open Biosystems: shNdn1 (TRCN0000103780), shNdn2 (TRCN0000103781), shNdn3 (TRCN0000103782). shGFP from Open Biosystems (RHS4459) was used as control vector. The Virapower lentivirus expression system (Invitrogen) allowed us to deliver genes of interest or shRNA in mixed populations (NIH and NIHLT). Briefly, the vector of interest was cotransfected with a packaging mix in 293FT. The supernatant was collected after three days and was either used fresh or concentrated. Infections were done overnight in the presence of polybrene, and puromycin selection was applied 48 hrs later.

### RNA and Proteins extractions

RNA was extracted directly from 80% confluent 100 mm petri dishes with TRIzol™ reagent (Gibco/BRL, Life Technologies Inc.). RNAs used in microarray experiments were further purified with QIAGEN Rneasy columns. Total proteins were extracted from 80% confluent 100 mm plates in buffer containing: 50 mM Tris HCl, pH 7.4, 150 mM NaCl, 1 mM EDTA, 1% TRITON| X-100, protease inhibitor Cocktail (Complete Protease Inhibitor cocktail Tablets, Roche), NaF and NaOV.

### Microarray analysis

Biotinylated hybridization targets were prepared from total RNA as described [101]. Affymetrix arrays Mu74a were used in experiment 1 to hybridize cRNA from the parental NIH3T3 population and from clones NIHLT11, NIHLT47, NIHLT40, NIHLT50. Arrays Mu74a2 were used in experiment 2 to hybridize NIHcM, an untransfected NIH3T3 sub-clone, and clones NIHLT7, NIHLT12, NIHLT32, NIHLT33, NIHLT46, and NIHLTGFPA (expressing a PyLT eGFP fusion protein). Gene expression levels were calculated for each EST from the scanned image by the Affymetrix GeneChip software algorithm. After normalization to the total average intensity, all probe sets whose expression levels were below 50 were raised to 50. The ∼25% of probes on the Mu74a arrays corresponding to Affymetrix synthesis errors were removed from both experiments for subsequent analysis. Based on data acquired by Northern blots analysis, individual microarray datasets were pooled into two groups, a first group containing samples with clearly detectable PyLT expression (NIHLT11, NIHLT32, NIHLT40, NIHLT46, NIHLT47, and NIHLT50), and a second group of samples lacking PyLT expression (NIH3T3, NIHcM, NIHLT7, and NIHLT12). Groups were compared against each other to detect significant differences in gene expression (Fold change of more than 50% increase/decrease at a *P* value of 0.02 or better, see Table S1). The candidate genes modulated at the transcriptional level by PyLT were reordered using GENESPRING™ to identify a group of genes that have an expression pattern closely matching PyLT expression levels (Table S2).

### Candidate gene expression validation

Northern blot analysis has been described elsewhere [18]. Radiolabeled probes were generated from RIKEN full-length cDNA. A subset of Northern blots were hybridized and revealed using Dig-labeled 1 kb cDNA probes according to the manufacturer's instructions (ROCHE Diagnostics).

### Western blotting and immunocytochemistry

The following antibodies were used for western blotting: Necdin (07-565), GFP (JL-8, 8371-2), p21 (F-5, sc-6246), p53 (Ab-1, clone PAb-240), HSP60 (N-20, sc-1052), Actin (AC-15, ab6276). The polyclonal antibody specific to PyLT (Mm1a) was produced by injection of PyB4a to form ascites in BN rat cells [102]. All HRP-conjugated secondary antibodies were purchased from Santa Cruz. Immunocytochemistry was performed on formaldehyde fixed cells with the DakoCytomation kit according to the manufacturer's protocol. p53 Pab240 antibody was used to detect non-functional p53 in the native form.

### Cell cycle analysis and proliferation

For p53 activation, 5 µM or 10 µM of nutlin-3 (Sigma) was used, with DMSO being used for untreated control. Flow cytometric analyses were performed to characterize cell cycle profiles. Approximately 1×10^5^ cells were seeded in 6-well plates and treated 24 hours later for the indicated period of time. Cells and medium were collected and centrifuged. Cells were fixed and stained with propidium iodide. The use of nutlin-3 mimics the overexpression of p53 as it causes the release of p53 from mdm2, which results in its accumulation [43]. Nutlin-3, like p53 overexpression, has been described to induce growth arrest in both the G1 and G2 phase [103], [104], [105]. Accordingly, we present FACS data as a ratio of arrested cells (G1+G2 phases) over proliferating cells (S phase). Cell proliferation reagent WST-1 (Roche) was used to follow growth arrest in NIH and NIHLT treated with nutlin-3. 5×10^3^ cells were plated in 96-well plates and treated 24 hrs later for a period of 48 hrs. Growth arrest is the difference between proliferation of untreated cells and proliferation of treated cells (O.D. untreated – O.D. treated). This result was normalized to growth arrest with the internal control NIH.

### Quantitative Real-Time PCR

Total RNA was extracted with TRIzol® reagent as described by the manufacturer and the quantity and quality were determined with the Agilent 2100 Bioanalyzer and NanoDrop. cDNA was generated using the QuantiTect® Reverse Transcription Kit (Qiagen). For Q-PCR, SYBR Green PCR Master Mix (Qiagen) was used for cDNA labeling and was performed with the Rotor-Gene 3000 Real-Time PCR Detection System (Corbett Life Sciences). The Pfaffl analysis method [106] was applied to data generated by Q-PCR. Primer sequences were from RTprimerDB [107] and are available upon request. For the ovarian cancers study, tumor samples were collected through the Division of Gynecologic Oncology at the Centre hospitalier de l'Université de Montréal (Hôpital Notre-Dame). We focused on samples of serous histopathology obtained from chemotherapy naïve patients. Experiments on ovarian tumor tissues were done twice in duplicate for each sample. Comparative gene expression analysis in ovarian samples was performed using ERK1 as an internal control based on previous results [108].

## Supporting Information

Table S1 **Microarrays analysis containing 194 genes modulated by PyLT expression in NIH3T3 (1,5 folds,** ***P*** **≤0.02).**
(XLS)

Table S2 **PyLT-mediated changes in gene expression profile in NIH3T3 cells.** A partial listing of PyLT modulated genes representing candidates whose expression variation correlates with the level of PyLT mRNA.
(XLS)

Figure S1 **PyLT expression and candidates selection.** (A) Western blot analysis of PyLT expression in selected clones used for microarrays analysis. The PyLT row represents the expression levels of PyLT protein in all clones (note that like the mRNA in Figure 1, PyLT protein in clone NIHLT33 is only detected on long exposures). (B) Genespring software representation of gene expression from candidates whose expression correlates with the level of PyLT, with emphasis on Necdin expression. PyLT mRNA expression levels by Northern blot analysis are presented below.
(TIF)

Figure S2 **Necdin induces growth arrest in short term experiment.** Overexpression of Necdin caused growth inhibition in three different experiments (A) Proliferation curve of NIH and NIHLT cells two weeks after transduction with Necdin or control vector. (B) Cellular proliferation assessed by colorimetric BrdU ELISA Kit (ROCHE) in the same population. (C) Proliferation monitored by Wst-1 assays on NIH3T3 cells transiently transfected with Necdin or control vector.
(TIF)

Figure S3 **Effects of Nutlin-3 stimulation in NIH population.** (A) Nutlin-3 stimulation of NIH cells induced an increase in p53 protein levels accompanied by an increase of its target gene p21 (B) Flow cytometry analysis of NIH and NIH shNdn1 to 3 treated with nutlin-3 showed no significant variation in growth arrest. (C) Wst-1 colorimetric essay on the same populations after 48 hrs of nutlin-3 treatment revealed increased sensitivity to growth arrest in Necdin-depleted NIH cells. Graph represents differences between treated and untreated cells normalized according to NIH control. (** *P*<0.01, t-test) (D) Q-PCR for p21 expression upon nutlin-3 stimulation (24 hrs) or control DMSO, in NIH and NIHLT cells overexpressing Necdin or Vector. (E) Protein levels in NIHLT cells containing shNdn or control 48 hrs after nutlin-3 stimulation.
(TIF)

Figure S4 **Nutlin-3 stimulation of NIH and NIHLT populations induces Necdin mRNA in a p53-dependent manner.** (A–B) Necdin was induced in a dose-dependent manner with nutlin-3 treatment in (A) NIH and (B) NIHLT. (C) Inactivation of p53 by transduction of NIH with GSE22 inhibited Necdin induction by nutlin-3. Relative expression by Q-PCR analysis according to GAPDH.
(TIF)
