# Supplementary figures and images for: Necdin, a p53-Target Gene, Is an Inhibitor of p53-Mediated Growth Arrest
Source: PLoS One. 2012 Feb 15;7(2):e31916. doi: 10.1371/journal.pone.0031916 (PMC3280226; doi:10.1371/journal.pone.0031916)

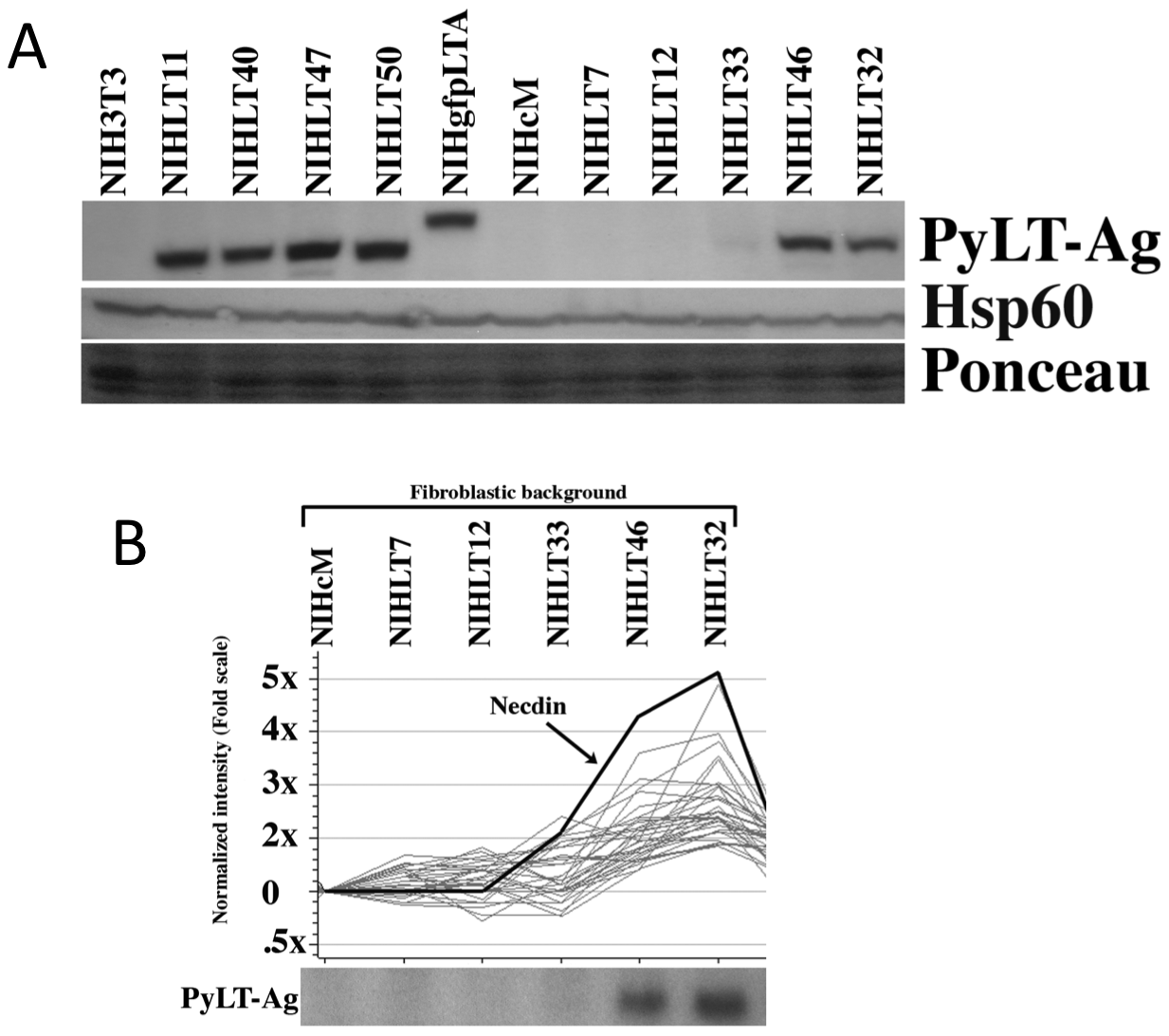

Supplement: Figure S1 — PyLT expression and candidates selection. (A) Western blot analysis of PyLT expression in selected clones used for microarrays analysis. The PyLT row represents the expression levels of PyLT protein in all clones (note that like the mRNA in Figure 1, PyLT protein in clone NIHLT33 is only detected on long exposures). (B) Genespring software representation of gene expression from candidates whose expression correlates with the level of PyLT, with emphasis on Necdin expression. PyLT mRNA expression levels by Northern blot analysis are presented below. (TIF) [file pone.0031916.s003.tif]

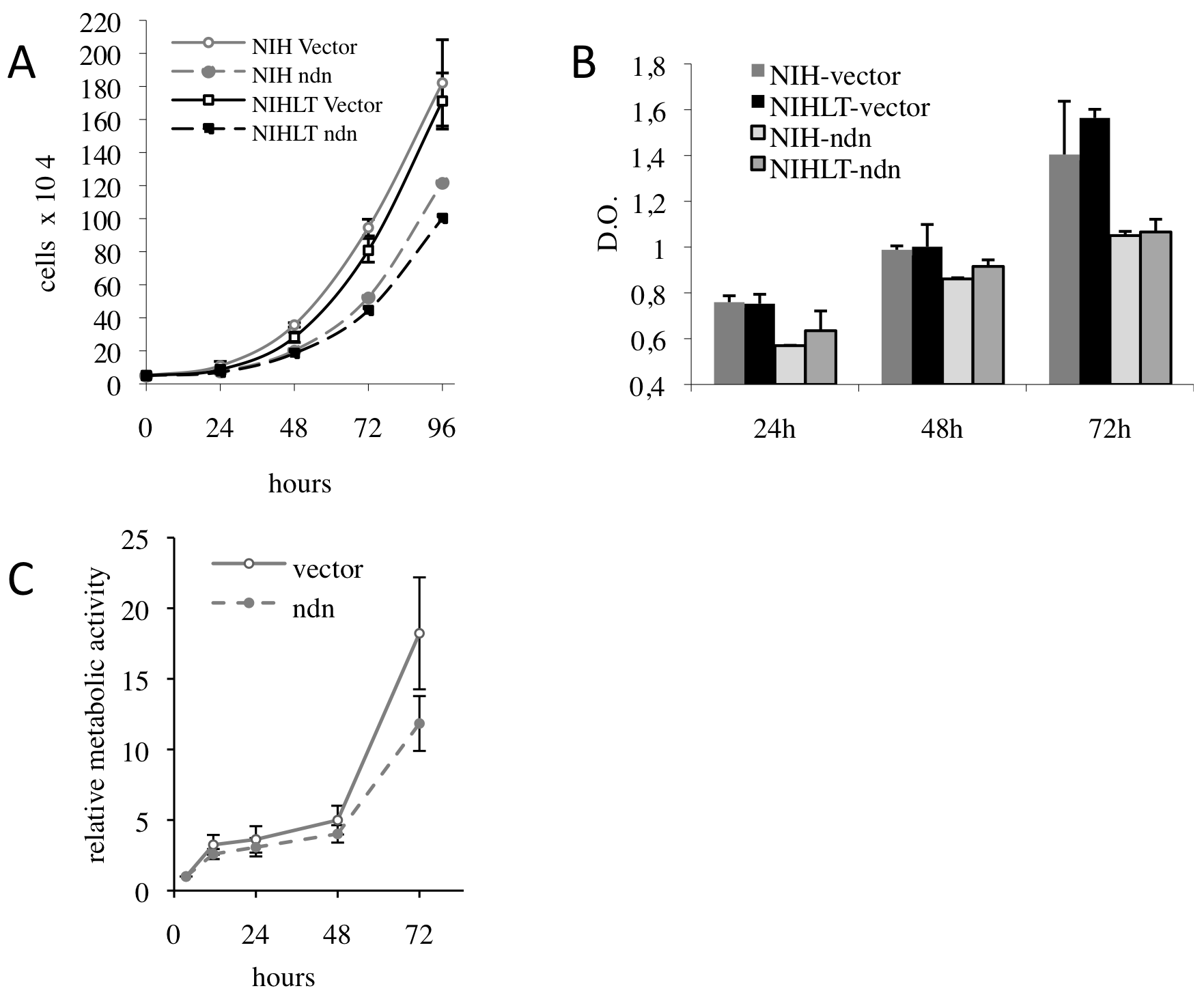

Supplement: Figure S2 — Necdin induces growth arrest in short term experiment. Overexpression of Necdin caused growth inhibition in three different experiments (A) Proliferation curve of NIH and NIHLT cells two weeks after transduction with Necdin or control vector. (B) Cellular proliferation assessed by colorimetric BrdU ELISA Kit (ROCHE) in the same population. (C) Proliferation monitored by Wst-1 assays on NIH3T3 cells transiently transfected with Necdin or control vector. (TIF) [file pone.0031916.s004.tif]

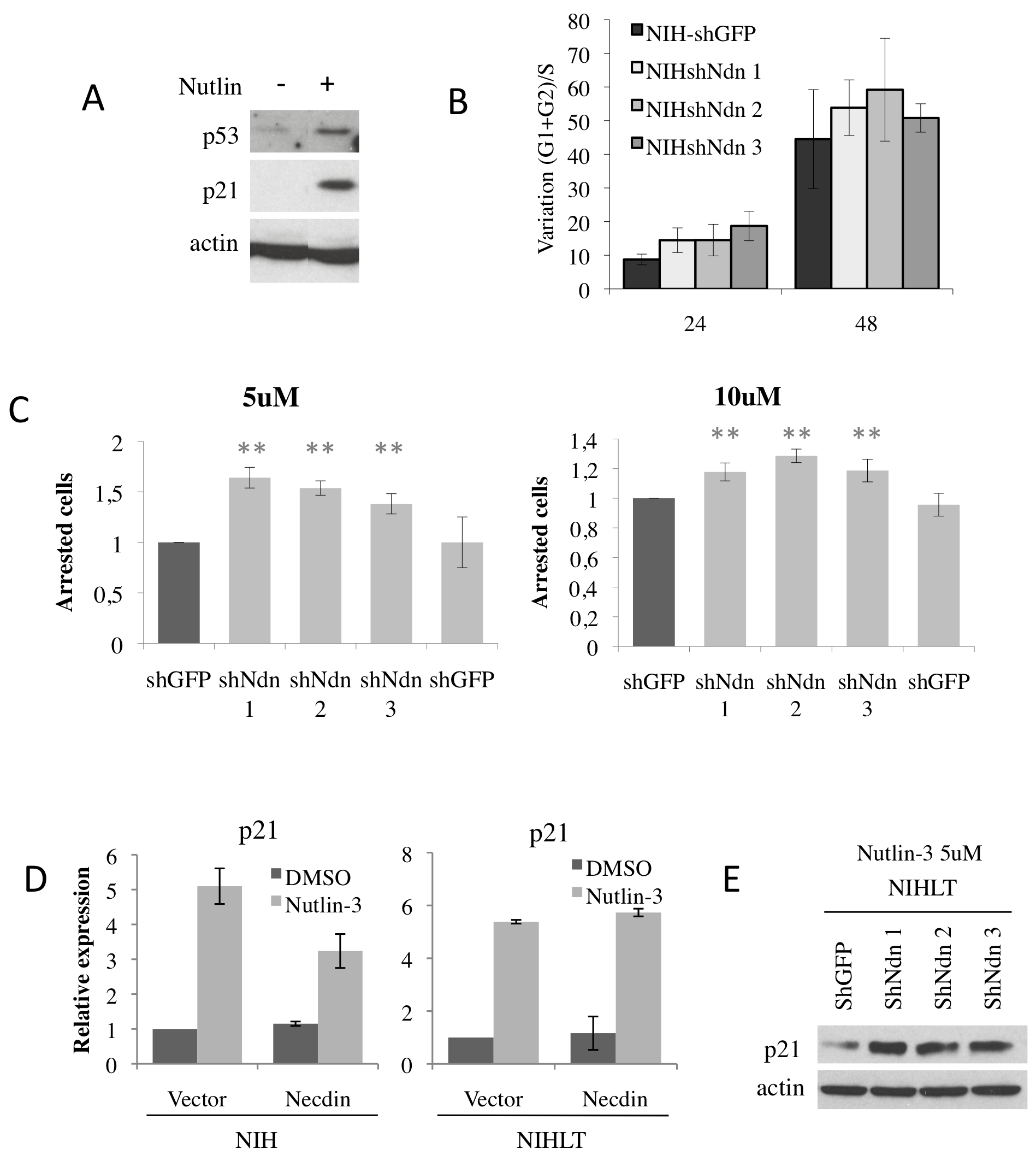

Supplement: Figure S3 — Effects of Nutlin-3 stimulation in NIH population. (A) Nutlin-3 stimulation of NIH cells induced an increase in p53 protein levels accompanied by an increase of its target gene p21 (B) Flow cytometry analysis of NIH and NIH shNdn1 to 3 treated with nutlin-3 showed no significant variation in growth arrest. (C) Wst-1 colorimetric essay on the same populations after 48 hrs of nutlin-3 treatment revealed increased sensitivity to growth arrest in Necdin-depleted NIH cells. Graph represents differences between treated and untreated cells normalized according to NIH control. (** P<0.01, t-test) (D) Q-PCR for p21 expression upon nutlin-3 stimulation (24 hrs) or control DMSO, in NIH and NIHLT cells overexpressing Necdin or Vector. (E) Protein levels in NIHLT cells containing shNdn or control 48 hrs after nutlin-3 stimulation. (TIF) [file pone.0031916.s005.tif]

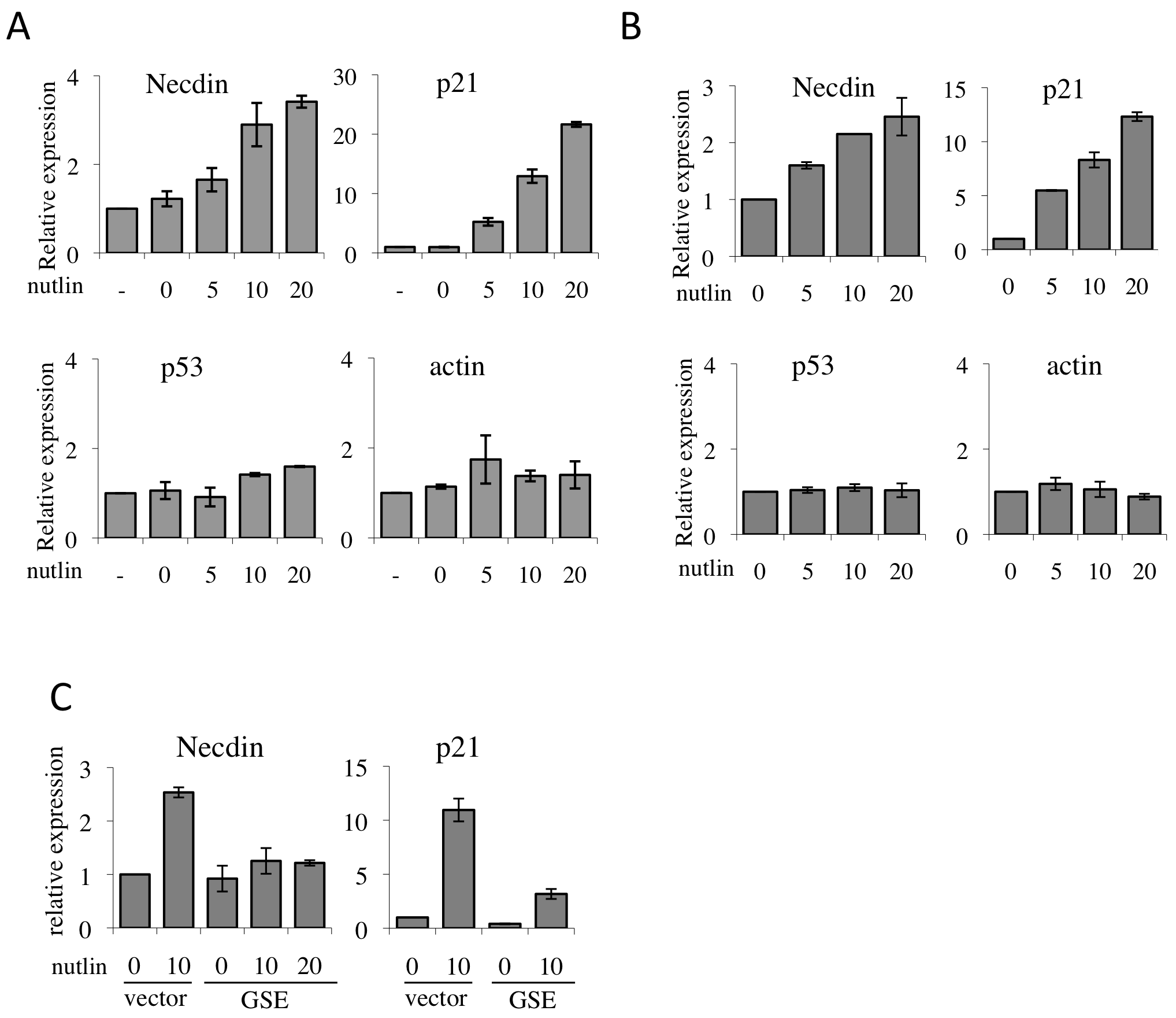

Supplement: Figure S4 — Nutlin-3 stimulation of NIH and NIHLT populations induces Necdin mRNA in a p53-dependent manner. (A–B) Necdin was induced in a dose-dependent manner with nutlin-3 treatment in (A) NIH and (B) NIHLT. (C) Inactivation of p53 by transduction of NIH with GSE22 inhibited Necdin induction by nutlin-3. Relative expression by Q-PCR analysis according to GAPDH. (TIF) [file pone.0031916.s006.tif]
